# Optimal treatment for Spinal Cord Injury associated with cervical canal Stenosis (OSCIS): a study protocol for a randomized controlled trial comparing early versus delayed surgery

**DOI:** 10.1186/1745-6215-14-245

**Published:** 2013-08-07

**Authors:** Hirotaka Chikuda, Hiroshi Ohtsu, Toru Ogata, Shurei Sugita, Masahiko Sumitani, Yurie Koyama, Morio Matsumoto, Yoshiaki Toyama

**Affiliations:** 1 Department of Orthopaedic Surgery, Faculty of Medicine, The University of Tokyo, 7-3-1 Hongo, Bunkyo-ku, Tokyo 113-8655, Japan; 2 Department of Clinical Trial Data Management, Graduate School of Medicine, The University of Tokyo, 7-3-1 Hongo, Bunkyo-ku, Tokyo 113-8655, Japan; 3 Department of Medical Engineering, Faculty of Medicine, The University of Tokyo, 7-3-1 Hongo, Bunkyo-ku, Tokyo 113-8655, Japan; 4 Rehabilitation for the Movement Functions, Research Institute, National Rehabilitation Center for Persons with Disabilities, Saitama 359-8555, Japan; 5 Department of Orthopaedic Surgery, School of Medicine, Keio University, 35 Shinanomachi, Shinjuku, Tokyo 160-0016, Japan

**Keywords:** Spinal cord injury, Surgery, Timing, Canal stenosis, Ossification of the posterior longitudinal ligament, Spondylosis, Spinal fracture, Bone injury

## Abstract

**Background:**

The optimal management of acute cervical spinal cord injury (SCI) associated with preexisting canal stenosis remains to be established. The objective of this study is to examine whether early surgical decompression (within 24 hours after admission) would result in greater improvement in motor function compared with delayed surgery (later than two weeks) in cervical SCI patients presenting with canal stenosis, but without bony injury.

**Methods/design:**

OSCIS is a randomized, controlled, parallel-group, assessor-blinded, multicenter trial. We will recruit 100 cervical SCI patients who are admitted within 48 hours of injury (aged 20 to 79 years; without fractures or dislocations; American Spinal Injury Association (ASIA) grade C; preexisting spinal canal stenosis). Patients will be enrolled from 36 participating hospitals across Japan and randomly allocated in a 1:1 ratio to either early surgical decompression (within 24 hours after admission) or delayed surgery following at least two weeks of conservative treatment. The primary outcomes include: 1) the change from baseline to one year in the ASIA motor score; 2) the total score of the Spinal Cord Independence Measure and 3) the proportion of patients who are able to walk without human assistance. The secondary outcomes are: 1) the health-related quality of life as measured by the Medical Outcomes Study Short Form 36 and the EuroQol 5 Dimension; 2) the Neuropathic Pain Symptom Inventory and 3) the walking status as evaluated with the Walking Index for Spinal Cord Injury II. The analysis will be on an intention-to-treat basis. The primary analysis will be a comparison of the primary and secondary outcomes one year after the injury.

**Discussion:**

The results of this study will provide evidence of the potential benefit of early surgical decompression compared to the current ‘watch and wait’ strategy.

**Trial registration:**

UMIN000006780; NCT01485458

## Background

Acute cervical spinal cord injury (SCI) is one of the most devastating conditions, and can lead to paralysis, sensory impairment and bowel, bladder and sexual dysfunction. In addition, patients frequently suffer from intractable pain caused by neural damage. Individuals with cervical canal stenosis are known to develop cervical SCI even after minor trauma. Cervical canal stenosis may be congenital, but often results from degenerative conditions, such as spondylosis. The SCI patients with canal stenosis are mostly elderly, and usually present with incomplete SCI without bone injury, such as spinal fracture or dislocation. This subgroup of patients has been steadily increasing as the society ages and currently accounts for over 60% of cervical SCIs in Japan [1].

The clinical outcome of patients with incomplete SCI has been considered to be favorable, since patients usually show spontaneous neurologic recovery to some extent. However, the neurological prognosis varies greatly among patients; about half of ASIA C patients remain non-ambulatory six months after the injury [2]. In particular, the clinical outcomes of elderly patients are often suboptimal [3,4]. Therefore, a therapeutic option that leads to a better clinical outcome is urgently needed.

Controversy exists with regard to the efficacy of surgical decompression in the treatment of cervical SCI with preexisting canal stenosis [5,6]. The role of surgery remain unclear, especially in the absence of instability of the cervical spine [7], thus resulting in a significant difference in practice between institutions. A common approach to treating these patients has been to rule out acute instability and then observe the patients’ spontaneous neurological recovery until they achieve a neurological plateau, and only then consider the possibility of surgical decompression, weeks after the initial injury [6]. Our previous retrospective multicenter study showed that the time from injury to surgery was approximately two weeks (median 13.5 days) [8].

The main drawback of this ‘watch and wait’ strategy is that a potential therapeutic window in the acute phase might be missed. The current concept of the pathophysiology of SCI classifies the spinal damage into two stages: primary injury and secondary injury [9]. The primary injury results from the mechanical forces delivered to the spinal cord at the time of the trauma. Secondary injury is a cascade of pathophysiological events including edema, ischemia, inflammation and apoptosis following the initial impact, which develops within minutes to hours following the trauma. There is a growing body of evidence from preclinical or animal studies that early surgical decompression alleviates ‘secondary injury’ and thus results in enhanced neurological and functional recovery [5].

Although numerous studies have been performed to examine the potential benefit of early surgery, the results of these prior clinical studies were mixed, and failed to provide robust support for the hypothesis that early surgery leads to improved outcomes. One small randomized trial of 42 patients showed no benefit to early (< 72 hours) decompression [10]. On the other hand, a meta-analysis of case series showed that early (< 24 hours) decompression was associated with better outcomes compared to both delayed (> 24 hours) and conservative treatment [11]. The results of STASCIS, one of the largest prospective studies of 313 patients, were also in favor of early surgery [12]. The authors of that study reported that early surgery, within 24 hours after injury, is associated with an improved neurological outcome, defined as at least a two grade ASIA Impairment Scale (AIS) improvement at the six-month follow-up examination. However, the difference in the chance of experiencing a one grade AIS improvement between early versus late surgery was not statistically significant.

With such conflicting information in the literature and a lack of high-quality evidence, it remains unclear whether early surgical decompression would result in better neurological and functional recovery. To address this issue, we launched the OSCIS study (Optimal treatment for Spinal Cord Injury associated with cervical canal Stenosis), a randomized, controlled, multicenter trial, in which we will compare the two strategies: early surgery within 24 hours after admission and delayed surgery following at least two weeks of conservative treatment.

## Methods/design

### Trial design

**Figure 1:**
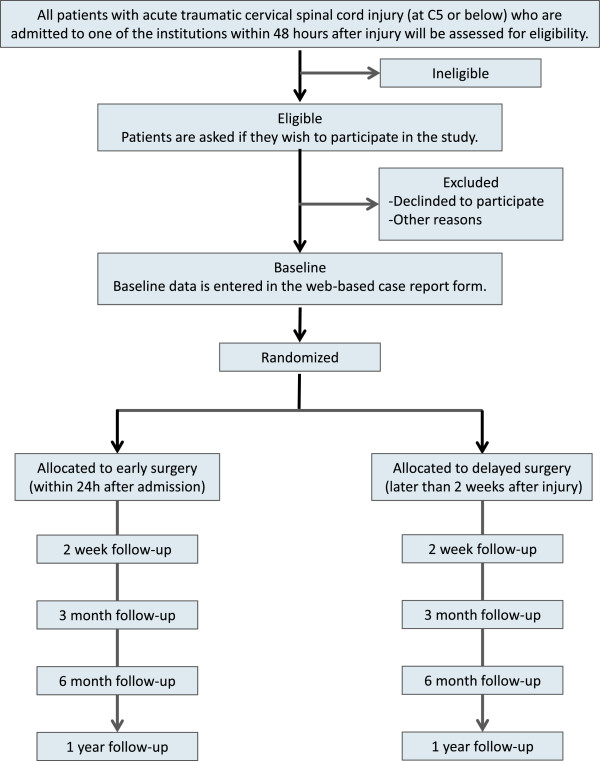
Study flowchart.

The OSCIS study is a randomized, controlled, parallel-group, assessor-blinded, multicenter study. Patients will be randomly allocated to undergo either early surgery or delayed surgery. The aim of this study is to test the hypothesis that early surgery (within 24 hours after admission) will lead to greater improvements in the motor function compared to delayed surgery (later than two weeks after injury) in patients with acute cervical SCI associated with canal stenosis. The flowchart shown in Figure 1 provides a visual description of the study.

### Participants

Subjects will be recruited from 36 hospitals in Japan. The list of the participating hospitals with approval from local ethical boards is available as Additional file 1. We will screen all patients with acute traumatic cervical spinal cord injury (at C5 or below) who are admitted to one of the institutions within 48 hours after the injury. The diagnosis of cervical spinal cord injury will be made on the patient’s history, including physical and neurological examinations, and the results of imaging studies, including plain radiographs, magnetic resonance imaging (MRI) and computed tomography (CT).

#### Inclusion criteria

Subjects will be eligible for inclusion if they satisfy the following inclusion criteria:

•aged 20 to 79 years

•without bone injury (spinal fracture or dislocation)

•American Spinal Injury Association (ASIA) impairment Grade C

•cervical canal stenosis due to preexisting conditions, such as spondylosis and ossification of the posterior longitudinal ligament (OPLL)

The presence of cervical canal stenosis will be confirmed by physicians based on the MRI findings obtained on admission. The presence of OPLL will be determined by using plain radiographs or CT. The thickness of the OPLL must be 20% or more of the spinal canal.

#### Exclusion criteria

Subjects will be excluded from enrollment if they meet any of the following conditions:

•unstable medical status

•unable to undergo surgery within 24 hours after admission

•impaired consciousness or mental disorder that precludes neurological examination

•difficulty in obtaining informed consent in Japanese

### Randomization

We will adopt the web-based allocation system using the University Medical Information Network (UMIN), which is one of the data centers that run as a public institution in Japan. By entering the information about the patient, investigators will be able to know the allocation results immediately.

The allocation table, which was created by stratified block randomized by the trial statistician, is registered in the UMIN. The block size is concealed to all investigators involved in this study. We have adopted stratification factors as follows:

•the presence of ossification of the posterior longitudinal ligament (OPLL) (yes/no)

•implementation of high-dose methylprednisolone treatment according to the NASCIS2 protocol (yes/no)

•preexisting gait disturbance due to myelopathy

•degree of canal compromise (50% or more/less than 50% canal compromise)

Preexisting gait disturbance due to myelopathy will be determined by the attending spine surgeon before randomization, based on thorough patients’ history and available medical record. Gait disturbance attributable to other causes (for example, trauma, osteoarthritis, and paralysis after stroke) will be excluded.

Presence of severe canal compromise (50% or more canal compromise) will be assessed by the attending spine surgeon based on mid-sagittal MR images obtained at admission. For patients presented with OPLL, mid-sagittal reconstruction CT images or plain radiographs of the cervical spine will be used to calculate the degree of canal compromise.

### Interventions

Patients will be randomly allocated to undergo either early surgery or delayed surgery.

#### Early surgery

Patients allocated to early surgery will undergo surgery within 24 hours after admission. The time when they enter the operating room will be used as a reference. The principal goal of surgery is to achieve decompression of the spinal cord. The choice of anterior or posterior approach will be left to the surgeon’s discretion. The use of spinal instrumentation will be permitted when needed. The surgery will be performed by or under supervision of a board-certified orthopedic surgeon. The details of the surgical treatment and any perioperative adverse events will be recorded in a web-based predefined form. All patients will receive intensive rehabilitation tailored to the individual and injury-specific factors immediately after surgery.

#### Delayed surgery

Patients allocated to the delayed surgery group will receive conservative treatment consisting of early mobilization and intensive rehabilitation for at least two weeks after the injury. Surgical decompression will be performed by the same team as in the early surgery group at any time later than two weeks after the injury when the physician thinks the timing is appropriate. Physicians will be allowed to treat patients non-surgically as long as the patients can achieve independent ambulation.

#### Other treatments

Apart from the surgical management, all patients will receive appropriate medical support, including permissive or induced hypertensive therapy (mean blood pressure > 85 mmHg) [13]. High-dose methylprednisolone will be used per the discretion of the treatment team according to the NASCIS-2 protocol [1,14,15]. The use or lack of high-dose methylprednisolone must be determined and entered into the web-based database prior to the randomization. Physicians will not be allowed to change or discontinue the administration of methylprednisolone after randomization.

### Primary and secondary outcomes

Participants will be evaluated two weeks, three months, six months and one year after randomization. Table 1 provides an overview of the outcomes that will be used in this study. Physicians and research nurses who are not involved in the patient’s care will assess the outcome at each follow-up examination before the patients see their doctors.

**Table 1:** The timeline of the outcome measures to be collected

		**Follow-up**			
	**Admission**	**2 weeks**	**3 months**	**6 months**	**1 year**
Visit	X^a^	X	X	X	X
Informed consent	X^a^				
Baseline clinical characteristics	X^a^				
Blood analyses	X^a^	X	X	X	X
Magnetic resonance imaging	X^a^				X
Computed tomography	X^a^				
Plain radiographs	X^a^	X			X
Neurological assessment including the ASIA motor score and ASIA impairment scale	X^a^	X	X	X	X
Evaluation of adverse events					
SCIM version 3		X	X	X	X
WISCI II		X			X
SF-36		X			X
EQ-5D		X	X	X	X
NPSI		X			X

X^a^: obtained prior to enrollment; ASIA: American Spinal Injury Association; EQ-5D: EuroQol 5 Dimension; NPSI, Neuropathic Pain Symptom Inventory; SCIM: Spinal Cord Independence Measure; SF-36: Medical Outcomes Study Short Form 36; WISCI: Walking Index for Spinal Cord Injury.

#### Primary outcomes

The primary outcome is a recovery in motor function one year after injury. The assessment will include: 1) the change from baseline to one year after the admission in the ASIA motor score; 2) the total score of the Spinal Cord Independence Measure (SCIM) version 3 and 3) the proportion of patients who regained the ability to walk 100 meters without human assistance.

The ASIA motor score is a 100-point score based on ten pairs of key muscles, each given a five point rating. The SCIM is a validated 100-point disability scale developed specifically for patients with SCI, with an emphasis on daily tasks grouped into three subscales: self-care (20 points), respiration and sphincter management (40 points) and mobility (40 points) [16-18].

#### Secondary outcomes

The secondary outcomes will include: 1) the health-related quality of life as measured by the Medical Outcomes Study Short Form 36 (SF-36) [19,20] and the EuroQol 5 Dimension (EQ-5D) [21]; 2) the neuropathic pain at the injured level and below as assessed by the Neuropathic Pain Symptom Inventory (NPSI) [22] and 3) the walking status as evaluated with the Walking Index for Spinal Cord Injury (WISCI) II [23].

The scores on the SF-36 will be used as a generic measure of the patient health status. The SF-36 comprises eight single subscale scores associated with physical and mental health.

The NPSI is a self-questionnaire specifically designed to evaluate the different symptoms of neuropathic pain. It includes 12 items, each of which is quantified on a (0 to 10) numerical scale. The pain associated with SCI is classified into two categories: at-level pain and below-level pain. Participants will be asked to complete the NPSI separately for pain in the upper extremities (at-level pain) and in the trunk and lower extremities (below-level pain). The WISCI II is a valid 21-level hierarchical scale of walking based on physical assistance, the need for braces and devices, with an ordinal range from 0 (unable to walk) to 20 (walking without assistance for at least 10 meters).

### Adverse events

The occurrence of pre-specified adverse events will be also assessed. Adverse events will be gathered from patients themselves and from the patient record review. The *a priori* defined adverse events are: worsening of paralysis in the upper extremities, worsening of paralysis in the lower extremities, reoperation, use of a respirator (more than one week), tracheostomy, sepsis, pneumonia, acute respiratory distress syndrome, atelectasis, other respiratory complications, wound infection (superficial), wound infection (deep), urinary tract infection, other infections, gastrointestinal bleeding, peptic ulcer, ileus, acute myocardial infarction, other cardiac events, pulmonary embolism, cerebrovascular complication, liver dysfunction/disease, renal dysfunction/disease, delirium, depression, other complications and death.

### Sample size

For this exploratory trial, the sample size was determined primarily based on feasibility. We assumed that it is feasible to enroll approximately 100 patients (50 patients per group) during the planned study period. As there is no valid data to indicate the optimal endpoint to evaluate the neurological and functional recovery of SCI patients, we selected three candidate endpoints as the primary endpoint: 1) the change from the baseline to one year after the admission in the ASIA motor score; 2) the proportion of patients who regained the ability to walk 100 meters without human assistance and 3) the total score of the Spinal Cord Independence Measure (SCIM) version 3.

We need 45 patients per group when the difference to be detected in the ASIA motor score between the groups is 12 points and the common standard deviation is 20. Additionally, we expect that the percentage of ambulatory patients one year after the injury will increase from 50% to 80%. To detect this difference, we need 39 patients for each group. With regard to the SCIM, there are few data that can be used as a basis for sample size calculation. For the reasons above, we set the sample size to be 50 patients per group. All calculations assume an 80% power at a two-tailed significance level of 0.05.

### Statistical methods

All analyses will be based on an intention-to-treat principal, and will be performed with two-sided *P*-values considered significant when they are below 0.05. For a detailed analysis, the statistician will make a statistical analysis plan before the data lock, as indicated below:

1) Primary endpoint:

•ASIA motor score

Calculate the difference one year after the baseline, and compare the two groups using a *t*-test

•The proportion of patients who regained the ability to walk

Calculate the rate of patients who can walk one year after the baseline, and compare the two groups using the chi-square test

•SCIM

Compare the differences in the SCIM after one year.

2) Secondary endpoint:

Compare the differences in the WISCI II, SF-36 and EQ-5D. For the SF-36, we plan to use only the total points, and not to compare each domain.

3) Safety:

We will compare the rates of adverse events between the groups. In particular, in patients that are moved out of the surgical standby group, we will compare the ratio of the occurrence of adverse events with those in the patients in the early operation group.

### Planned subgroup analyses

Predefined subgroup analyses will be performed in patients with or without OPLL. These will include high-dose methylprednisolone treatment, preexisting gait disturbance and severe canal compromise (> 50% canal compromise). Based on our previous study, we hypothesize that early surgical decompression will be beneficial in patients with preexisting gait disturbance and those with severe canal compromise.

### Ethical issues

The study protocol was approved by the local ethics committees of all participating hospitals and will be done in accordance with the Declaration of Helsinki. The study will be overseen by an independent safety monitoring board. All participants will give written informed consent before entry.

Ethical approval was obtained from all participating hospitals. The results will be disseminated via the usual scientific forums, including peer-reviewed publications and presentations at international conferences.

## Discussion

Despite intensive basic and clinical research, an effective treatment for cervical SCI has not been established. In the presence of preexisting canal stenosis, the role of surgical decompression and its optimal timing continue to be subjects of intense debate. Addressing the issue of the timing of surgical intervention is critical in that, if the timing of surgery has no effect on the patient’s outcome, then all patients can initially be treated non-surgically and surgery can be delayed for weeks or even months after the injury without compromising the patient’s recovery [6]. On the other hand, if early surgical decompression is proven to be beneficial, drastic changes in the medical service system, including logistics, should be made to ensure that all SCI patients receive early surgery.

In conducting clinical studies on SCI, the heterogeneity of the study population can be a major obstacle, especially in the acute phase. SCI patients vary greatly in the severity of paralysis and neurological prognosis. Clinical studies including patients with various degrees of neurological injury may have insufficient power. Therefore, in this study, we will focus on patients with ASIA C status. In a recent review, the consensus of experts was that it is reasonable to consider early surgical decompression in patients with profound neurologic deficit (ASIA C) and spinal canal stenosis without fracture or instability. On the other hand, those with a less severe deficit (ASIA D) can be treated with initial observation with surgery potentially performed at a later date [6]. We will exclude patients with ASIA B status, because these patients are often difficult to distinguish from ASIA A patients at the time of admission.

The information available regarding the window of opportunity or therapeutic window in human SCI is imprecise [24] and the definition of ‘early surgery’ has not yet been well established. Although the ideal cutoff time at which surgery provides potential neuroprotection is not known, the most intensively investigated times in the prior studies were 24 and 72 hours. In this study, we have adopted a cutoff at twenty-two hours after admission mainly for practical and logistic reasons. Twenty-four hours after admission is considered to be necessary and sufficient to safely perform the initial evaluation of patients and summon the operating team for emergency surgery. In this study, we adopted the time of admission as a reference, since the time of injury sometimes remains conjectural.

The OSCIS study is designed to provide evidence of the potential benefit of early surgical decompression over a wait-and-see strategy. We believe that the results of this trial will have a substantial impact on the management of cervical SCI.

## Trial status

The trial was registered in the UMIN register on 1 December, 2011. The first patient was randomized on 3 December, 2011. The trial is currently open for recruitment.

## Abbreviations

AIS: ASIA Impairment Scale; ASIA: American Spinal Injury Association; CT: Computed tomography; EQ-5D: EuroQol 5 Dimension; MRI: Magnetic resonance imaging; OPLL: Ossification of the posterior longitudinal ligament; SCI: Spinal cord injury; SCIM: Spinal Cord Independence Measure; SF-36: Medical Outcomes Study Short Form 36; UMIN: University Medical Information Network; WISCI: Walking Index for Spinal Cord Injury.

## Competing interests

The authors declare that they have no competing interests.

## Authors’ contributions

HC, HO, TO, SS, MS, MM, and YT participated in the conception and design of the study. YK will participate in the monitoring and quality control of the data. HC drafted the manuscript. All authors read, commented on and approved the manuscript.

## Supplementary Material

Additional file 1 List of participating hospitals with approval from local ethical boards (as of 6 August, 2013). Click here for file
