# Supplementary material for: Optimal treatment for Spinal Cord Injury associated with cervical canal Stenosis (OSCIS): a study protocol for a randomized controlled trial comparing early versus delayed surgery
Source: Trials. 2013 Aug 7;14:245. doi: 10.1186/1745-6215-14-245 (PMC3750661; doi:10.1186/1745-6215-14-245)
Supplement: Additional file 1 — List of participating hospitals with approval from local ethical boards (as of 6 August, 2013). [file 1745-6215-14-245-S1.docx]

List of participating hospitals with approval from local ethical boards (as of August 6, 2013)

1. The University of Tokyo
2. Osaka University
3. Hoshigaoka Koseinenkin Hospital
4. National Hospital Organization Osaka Minami Medical Center
5. Tokyo Metropolitan Bokutoh Hospital
6. Tokyo Metropolitan Tama Medical Center
7. Saitama Medical Center, Jichi Medical University
8. Saitama Medical Center, Saitama Medical University
9. Kurashiki Central Hospital
10. Wakayama Medical University
11. Imakiire General Hospital
12. Nihon University
13. Jichi Medical University
14. Japan Labour Health and Welfare Organization, Kanto Rosai Hospital
15. Japan Labour Health and Welfare Organization, Kansai Rosai Hospital
16. Hokkaido Chuo Rosai Hospital Sekison Center
17. Nagoya University
18. Kurume University
19. Hamamatsu University School of Medicine
20. St. Marianna University School of Medicine
21. Kyorin University
22. University of Toyama
23. Kagoshima University
24. Sendai Medical Center
25. Dokkyo Medical University
26. Keio University
27. Tokyo Medical and Dental University
28. Niigata City General Hospital
29. Chiba Aoba Municipal Hospital
30. Juntendo University Shizuoka Hospital
31. Yokohama Rosai Hospital
32. Kobe City Medical Center General Hospital
33. Kimitsu Chuo Hospital
34. Chiba University
35. Tokai University
36. Spinal Injuries Center
